# Local Methotrexate Injection Followed by Dilation and Curettage for Cesarean Scar Pregnancy: A Prospective Non-randomized Study

**DOI:** 10.3389/fmed.2021.800610

**Published:** 2022-01-21

**Authors:** Kai-Liang Tan, Yu-Mei Chen, Wei Zeng, Ying Meng, Li Jiang

**Affiliations:** Department of Obstetrics and Gynecology, Maternal and Child Health Hospital of Guangxi Zhuang Autonomous Region, Nanning, China

**Keywords:** cesarean scar pregnancy, methotrexate, local injection, uterine artery embolization, dilation and curettage

## Abstract

**Purpose:**

To evaluate the clinical effects and outcomes of local intra-gestational sac methotrexate injection followed by dilation and curettage for treatment of cesarean scar pregnancies (CSP).

**Method:**

This prospective non-randomized study was conducted on patients diagnosed with CSP between 2018 and 2020 at the Maternal and Child Health Hospital of Guangxi Zhuang Autonomous Region. Patients were categorized into two groups according to the treatments, i.e., local intra-gestational sac methotrexate injection followed by dilation and curettage (group A), and uterine artery embolization in combination with dilation and curettage (group B). The choices of treatment reflect the patients' decision after they thoroughly understood the benefits and risks of the two therapies. Clinical data were then collected and compared between these two alternatives.

**Results:**

Seventy-seven patients with CSP were enrolled in the study. Of this total, 41 vs. 36 were respectively categorized into group A and group B. Similar success rates were observed between these two groups (92.7 vs. 97.2%; RR = 27.362, 95% CI: 0.496–1.51E3, *p* = 0.106). However, the overall occurrence of complications in group A was significant lower when compared with group B (17.1 vs. 52.8%; RR = 0.236, 95% CI: 0.077–0.728, *p* = 0.012). Lower abdominal pain (unrelated to infection) and intrauterine adhesions were the two primary complications exhibited in group B of the present study, with rates of 38.9 and 22.2% respectively.

**Conclusions:**

Local intra-gestational sac methotrexate injection followed by dilation and curettage is an effective and safe treatment for CSP that also drastically reduces the risks of complications. Further multiple center randomized trials with large series are warranted to confirm these findings.

## Introduction

Cesarean scar pregnancy (CSP) is a consequent result of a previous cesarean section. CSP may cause uterine rupture, massive hemorrhaging, and potentially become life threatening (1–3). Its incidence is estimated to be from 1 in 2,216 up to 1,800 (1, 2). Recently, concern has been gradually increasing because of the climbing rate of CSP (4). Early diagnosis and appropriate treatment play an essential role in improving patients' outcome (2, 5–7). Transvaginal ultrasound is the primary method of diagnosing CSP (8). Although a variety of modalities for treatments of CSP have been reported recently, including systemic or local injection of methotrexate, hysteroscopy, dilation and curettage (D&C), surgical resection, and uterine artery embolization (UAE) (9–12), there is no consensus on the optimal treatment for patients who desire to maintain fertility (13). Uterine artery embolization is widely used and has been demonstrated to be effective in reducing the risk of bleeding while treating CSP (12), but it is associated with potential complications (14, 15). However, UAE is not widely available because of the required specialized equipment and procedures. In addition, physicians who would like to use UAE require additional training, which is especially onerous in some developing regions.

Our group's preliminary report suggests local intra-gestational sac methotrexate injection combined with D&C appears to be effective in the treatment of CSP (16). However, until now, no case-control study has been conducted to conclusively evaluate its safety and efficacy. Our group has conducted a prospective study to compare the therapeutic effects of local intra-gestational sac methotrexate injection and uterine artery embolization which are then combined with D&C.

## Materials and Methods

### Patients

The study, approved by the local Ethics Committee [ethic number: (2017-2)2], evaluates patients admitted for CSP at the Maternal and Child Health Hospital of Guangxi Zhuang Autonomous Region from January 2018 to December 2020. After a thorough discussion of the options, a treatment program was decided upon by patients after they had completely considered the benefits and risks of each therapy. Then they were classified into either local intra-gestational sac methotrexate injection followed by dilation and curettage (group A), or uterine artery embolization in combination with dilation and curettage (group B). Informed consent was obtained from each participant prior to treatment.

Cesarean scar pregnancy was diagnosed based on a patient's health history, positive pregnancy test and the findings of transvaginal ultrasound as the previously reported criteria (11). CSP is categorized into type I, type II and type III based on the location and shape of gestational tissue, blood flow features, and thickness of the myometrium at the incision site, according to the guideline provided by “Expert opinion of Diagnosis and Treatment of Cesarean Scar Pregnancy (2016)” issued by the Family Planning Group of Obstetrics and Gynecology Credit Association of the Chinese Medical Association (17). The diagnostic standards for CSP types has been described in a previous report (18).

The inclusive criteria were as follows: (1) gestational age ≤ 12 weeks, (2) patients with CSP exhibited no heavy vaginal bleeding prior to treatment, (3) all participants were hemodynamically stable, (4) hemoglobin was at least 90 g/L, and (5) patients had no contraindication for methotrexate. Patients who had any of these symptoms were excluded, including those with massive vaginal bleeding, unstable vital signs, acute pelvic inflammation, impaired renal function, impaired liver function, or clotting disorders.

### Local Intra-gestational Sac Methotrexate Injection Followed by Dilation and Curettage

Two primary steps are performed as previously reported: methotrexate injection under ultrasound guidance and the removal of retained products of conception (16). Access was obtained via the cervix to the gestational sac using transvaginal ultrasound guidance without administering anesthesia. Methotrexate (50 mg/m^2^) dissolved in 5 mL of saline was injected into the gestational sac. One week after the procedure, evaluations were conducted of serum β-HCG levels, liver and renal function, and complete blood cell counts as well as pelvic ultrasounds. A second dose of local methotrexate injection (50 mg/m^2^) was repeated one week after the original dose if serum β-HCG showed < a 15% decline.

When the serum β-HCG level decreased approximately to or <10,000 mIU/mL, a dilation and curettage (D&C) was performed under intravenous anesthesia and transvaginal ultrasound guidance as was reported in our previous study (16). In case of active bleeding, a 14# Foley's balloon catheter was inserted into the cesarean scar area and inflated with sterile water under ultrasound guidance. This catheter could then apply the necessary pressure to mitigate bleeding. The Foley's balloon catheter was withdrawn after 24 h.

### Uterine Artery Embolization in Combination With Dilation and Curettage

A right femoral artery puncture was performed. A 5F catheter was inserted and then bilateral internal iliac artery angiography was carried out. The uterine artery was selectively catheterized, perfused with 50 mg methotrexate and embolized with gelatin sponge particles (560–1,400 μm size). Uterine arteries were verified to be embolized according to the angiography. A dilation and curettage was performed under intravenous anesthesia and transvaginal ultrasound guidance 24 to 48 h after UAE. A 14# Foley's balloon catheter was inserted into the cesarean scar area to alleviate bleeding if active bleeding was occurring. The Foley balloon was withdrawn after 24 h.

### Follow Up and Outcomes Measures

After dilation and curettage, the serum β-HCG level was monitored weekly until it declined to normal range of (<3 mIU/mL). The patient's menstrual information was monitored and updated via remote interviews. A pelvic ultrasound scan was performed after menstrual recovery. The criteria for successful treatment were indicated by: (1) normalization of ultrasonographic findings and serum β-HCG, (2) no uterine rupture, (3) no conversion to surgical resection, (4) blood loss limited to 500 mL or less during operation and follow up, and (5) no required blood transfusion.

The proportion of patients successfully treated by each method will be measured as the primary outcome. Secondary outcomes are determined as follows: complication rate, duration of D&C, blood loss during D&C, time of ß-HCG normalization and rate of blood transfusion. Complications include lower abdominal pain (unrelated to infection), pelvic inflammation, excessive bleeding (Blood loss ≥ 200 ml), uterine rupture, intrauterine adhesion and side effects linked to methotrexate (nausea, vomiting, gastrointestinal ulcer, myelosuppression, impaired liver and renal function, et al). Intrauterine adhesions were evaluated by transvaginal ultrasound during follow up. Adhesions were shown by uneven echo of the endometrium and interruption of local echo of endometrium continuity. Hysteroscopic adhesiolysis was perform for patients with intrauterine adhesions who sought future childbearing or suffered from menstrual blood retention.

### Statistical Analysis

Demographic, clinical and therapeutic characteristics were compared between these two groups. Normally distributed data and skewed data were described by mean ± SD and median (25th, 75th), respectively. Independent sample-*t* tests and the Mann-Whitney test were used to compare the normally distributed data and skewed data respectively. Categorical data were presented as frequencies and percentages. Either the Chi-squared test or Fisher's Exact test was used to determine the categorical variable. Multivariate logistic regression analysis was used to adjust the potential confounding factors and analyze the effects of the treatment on outcome. The level of statistical significance was set at *p* < 0.05. Statistical analyses were performed using the SPSS version 16.0 software.

## Results

A total of 217 CSP patients were diagnosed in our hospital during this period. Of the 217, 140 were excluded. Seventy-seven patients were recruited into the study. Of these cases, 41 and 36 were respectively categorized into local intra-gestational sac methotrexate injection followed by dilation and curettage (group A), and uterine artery embolization in combination with dilation and curettage (group B). There were no statistically significant differences of age, number of previous CS, gestational age, type of CSP, fetal cardiac activity or thickness of uterine scar between the two groups. The median pretreatment serum ß-HCG was lower in group A, compared with group B (56,022 mIU/mL vs. 92,760 mIU/mL, *p*<0.001). The mean sac diameter was slightly smaller in group A than group B (2.2 ± 0.86 cm vs. 2.7±0.82 cm, *p* = 0.006) (Table 1).

**Table 1:** Demographic and clinical characteristic of patients with CSP.

**Variables**	**Group A**	**Group B**	***p***
	**(*n* = 41)**	**(*n* = 36)**	
Age (years)	34.1 ± 4.7	33.1 ± 3.9	0.336
Gestational ages (days)	53.98 ± 10.49	54.44 ± 9.50	0.839
Thickness of uterine scar (cm)	0.22 ± 0.08	0.20 ± 0.07	0.223
Mean sac diameter (cm)	2.21 ± 0.86	2.76 ± 0.82	0.006
Pretreatment serum ß-HCG			
median (25th, 75th) mIU/mL	56,022 (31,376, 72,344)	92,760 (58,076, 11,8837)	<0.001
Type of CSP			
I	6 (14.6%)	1 (2.8%)	0.087
II	31 (75.6%)	34 (94.4%)	
III	4 (9.8%)	1 (2.8%)	
Number of previous CS			
1	26 (63.4%)	18 (50%)	0.235
≥2	15 (36.6%)	18 (50%)	
Fetal cardiac activity			
Yes	29 (70.7%)	28 (74%)	0.482
No	12 (29.3%)	8 (26%)	

*CSP, cesarean scar pregnancy; CS, cesarean section; ß-HCG, serum beta human chorionic gonadotropin; Group A, local intra-gestational sac methotrexate injection followed by dilation and curettage; Group B, uterine artery embolization in combination with dilation and curettage*.

The duration of D&C, blood loss during D&C and blood transfusion rates were comparable between the two groups (Table 2). There was no significant difference in the success rates between group A and group B (Tables 2–4). However, the complication rate in group A was significant lower when compared with group B (17.1 vs. 52.8%; RR = 0.236, 95% CI: 0.077–0.728, *p* = 0.012). Lower abdominal pain unrelated to infection and intrauterine adhesions were the two primary complications exhibited in group B of the present study, with rates of 38.9 and 22.2% respectively. As no one suffered from lower abdominal pain unrelated to infection among group A, the OR and 95% CI value of group A compared with group B cannot be calculated by logistic regression analysis.

**Table 2:** Comparison of effects and outcomes between group A and group B.

**Variables**	**Group A**	**Group B**	***p***
	**(*n* = 41)**	**(*n* = 36)**	
Success rate (%)	92.7% (38/41)	97.2% (35/36)	0.618
Complication rate (%)	17.1% (7/41)	52.8% (19/36)	**0.001**
Duration of D&C (min)	15.6 ± 5.1	16.7 ± 5.8	0.457
Blood transfusion (%)	4.9% (2/41)	0 (0/36)	0.496
Blood loss during D & C			
median (25th, 75th) ml	10 (10, 40)	10 (5, 20)	0.063
Time of ß-HCG resolution			
after D&C (weeks)	3.4 ± 0.7	4.7 ± 1.5	<0.001

*β-HCG, serum beta human chorionic gonadotropin; D & C, dilation and curettage; Group A, local intra-gestational sac methotrexate injection followed by dilation and curettage; Group B, uterine artery embolization in combination with dilation and curettage. Bold values highlighted with its statistical significance*.

**Table 3:** Comparison of complications between group A and group B.

**Variables**	**Group A**	**Group B**	***p***
	**(*n* = 41)**	**(*n* = 36)**	
Lower abdominal pain (unrelated to infection)	0 (0/41)	14 (38.9%)	**<0.001**
Pelvic infected disease	1 (2.4%)	1 (2.8%)	1.0
Blood loss ≥ 200 ml	3 (7.3%)	1 (2.8%)	0.618
Intrauterine adhesion	2 (4.9%)	8 (22.2%)	**0.039**
Impaired liver function	1 (2.4%)	0 (0/36)	1.0

*Group A, local intra-gestational sac methotrexate injection followed by dilation and curettage; Group B, uterine artery embolization in combination with dilation and curettage. Bold values highlighted with its statistical significance*.

**Table 4:** Multivariate logistic regression analysis of the effects of local methotrexate injection on cesarean scar pregnancy compared with uterine artery embolization.

**Outcomes**	**B**	**S.E**.	**Wald**	***p***	**RR^**a**^**	**95%CI**
Successfully treatment	3.309	2.048	2.612	0.106	27.362	0.495–1.51E3
Complications	−1.442	0.573	6.324	0.012	0.236	0.077–0.728
Lower abdominal pain (unrelated to infection)^*^	−20.621	6.20E3	1.10E-5	0.997	–	–
Intrauterine adhesion	−2.392	1.027	5.427	0.020	0.091	0.12–0.684

*β-HCG, serum beta human chorionic gonadotropin; B, partial regression coefficient; S.E., standard error of partial regression coefficient; RR, relative risk; CI, confidence interval*.

a *Adjusted factors included pretreatment serum ß-HCG, mean sac diameter, fetal cardiac activity, thickness of uterine scar, and gestational ages*.

* *As no one suffered from low abdominal pain (unrelated to infection) among group A, the RR and 95% CI of group A compared with group B cannot be calculated by logistic regression analysis*.

The 14 patients with lower abdominal pain unrelated to infection were administered analgesics. One patient was administered with pethidine, while the other 13 were prescribed with tramadol hydrochloride. Four patients who sought continued childbearing were provided with hysteroscopy and adhesiolysis procedures. Two patients with pelvic infection experienced symptoms of fever and lower abdominal pain. Only one patients developed a side effect linked to methotrexate, which was impaired liver function. Neither uterine rupture nor mortality occurred in either group.

## Discussion

The aim of the current study is to evaluate clinical effects of local intra-gestational sac methotrexate injection with subsequent dilation and curettage (D&C) in the treatment of cesarean scar pregnancies. Statistics demonstrate that the local intra-gestational sac methotrexate injection method combined with D&C has achieved a high success rate. This is not significantly different from that of UAE combined with D&C through a prospective non-randomized cohort study.

Further attention is being paid to treatment options because CSP frequently causes subsequent severe morbidity (1, 2). Several approaches have been shown to be safe and feasible for the treatment of CSP (4). Methotrexate has been widely used in treating CSP, including systemic and local injection (9). Recently, evidence suggests that systemic methotrexate is not a suitable option for CSP because of its lower success rate and elevated risk of side effects (4). The advantages of local intragestational methotrexate to treat CSP has been previously described (19, 20). However, the medical treatment alone of intragestational methotrexate remains controversial (13, 21). A previous study reported that a local combined with systemic methotrexate without D&C has a success rate of 80.9% (22). The present study describes a combination treatment of local intra-gestational sac methotrexate injection followed by D&C, thus achieving an appreciably higher success rate of 92.7%. This result aligns with our previous reports (16). Additionally, the retained gestational tissue creates a potential risk of hemorrhaging, and it required two months to one year, to resolve spontaneously (1, 23). The D&C was performed in group A when serum β-HCG level decreased approximately to or <10,000 mIU/mL. This may result in a shorter time of ß-HCG resolution after D&C.

Uterine artery embolization (UAE) can profoundly reduce the risk of intraoperative hemorrhages and has been demonstrated to be an effective treatment for CSP (14, 24). In the present study, 97.2% of patients were treated successfully using UAE and D&C (group B). Its high efficacy was consistent with earlier reports (12). However, the frequency of complications was significantly higher in group B, compared with group A (52.8 vs. 17.1%, *p* = 0.001). Lower abdominal pain (unrelated to infection) and intrauterine adhesions were the two primary complications exhibited in group B in this study. Elevated rates of lower abdominal pain (11.2–15.1%) were also observed in previous studies (14, 25). This symptom may be related to uterine ischemia, which is a complication frequently expressed secondarily to UAE (26). There is no consensus on the pain management after UAE (26, 27). Tramadol hydrochloride also seemed effective for pain secondary to UAE in the present study. However, pelvic inflammatory disease should be differentiated with further examinations when patient develop corresponding symptoms, such as fever (28).

The rate of intrauterine adhesion was a high 22.2% in the present study and is in accordance with a previous report (29). Endometrial atrophy secondary to UAE may explain the higher risk of intrauterine adhesions (29, 30). Additionally, UAE may reduce menstrual blood volume and future pregnancy rates (14). Therefore, it is prudent to administer UAE to patients who desire future childbearing options. In addition, it is necessary to raise concerns about several other complications secondary to UAE, such as infectious disease, deep venous thrombosis, acute pulmonary embolism, and inadvertent embolization (31). UAE also increases the economic burden because of its specific instruments and procedure (7, 25), which may limit its broader use.

There is no consensus on the category of types of CSP and treatment options based on these types. According to the guide “Expert opinion of Diagnosis and Treatment of Cesarean Scar Pregnancy (2016)” issued by the Family Planning Group of Obstetrics and Gynecology Credit Association of the Chinese Medical Association, CSP is categorized into type I, type II and type III based on the location and shape of gestational tissue, blood flow features, and thickness of the myometrium at the incision site (17). Type II and III CSP were found to be associated with excessive intraoperative hemorrhaging in a previous study (7). In our study, four out of five cases with type III CSP had blood loss in excess of 200 ml during D&C. This includes three cases in group A who failed the primary treatment. Thus, it may be a disadvantageous option for type III CSP to undertake the approach of local intra-gestational sac methotrexate injection followed by D&C. It seems to indicate UAE or surgical resection may improve the outcome of these patients based on the current evidence (7, 25, 32).

To the best of our knowledge, this is the first report that compares the clinical effects and outcomes of local intra-gestational sac methotrexate injection and uterine artery embolization in conjunction with D&C in treating cesarean scar pregnancy. This report may provide clinical evidence for shaping future treatment guidelines for CSP. This study includes several drawbacks: Firstly, this is a non-randomized trial because of a low rate of CSP in the target facility, which may produce selection bias. For example, the size of sac and pretreatment serum ß-HCG levels are statistically different between these two groups, which may potentially produce an impact on the results (33). However, the outcomes were not affected after factors with differences were adjusted using logistic regression analyses. Secondly, long-term effects such as reproductive outcomes are not addressed because of the short-term nature of the study. Additionally, the defect of uterine scaring was not considered. This leaves patients still at risk of CSP in future pregnancies.

In summary, the results of this study suggest that local intra-gestational sac methotrexate injection in association with D&C is a desirable option for managing CSP, providing a reduced complication rate as compared with uterine artery embolization and D&C. Further randomized multiple center trials with large sample sizes are essential to confirm these results.

## Data Availability Statement

The original contributions presented in the study are included in the article/supplementary material, further inquiries can be directed to the corresponding author/s.

## Ethics Statement

The studies involving human participants were reviewed and approved by Ethics Committee of Maternal and Child Health Hospital of Guangxi Zhuang Autonomous Region. The patients/participants provided their written informed consent to participate in this study.

## Author Contributions

K-LT conceived and designed the study, analyzed clinical data, and drafted the manuscript. Y-MC collected data and supervised the protocol development. WZ collected data. YM and LJ were responsible for drafting and editing the manuscript. All authors contributed equally to the revision of the manuscript and approved the final manuscript.

## Funding

This work was supported by the Guangxi Health Commission Department Research Program (Grant No: Z20170769).

## Conflict of Interest

The authors declare that the research was conducted in the absence of any commercial or financial relationships that could be construed as a potential conflict of interest.

## Publisher's Note

All claims expressed in this article are solely those of the authors and do not necessarily represent those of their affiliated organizations, or those of the publisher, the editors and the reviewers. Any product that may be evaluated in this article, or claim that may be made by its manufacturer, is not guaranteed or endorsed by the publisher.
